# Increasing Susceptibility of Drug-Resistant *Candida albicans* to Fluconazole and Terbinafine by 2(5*H*)-Furanone Derivative

**DOI:** 10.3390/molecules25030642

**Published:** 2020-02-02

**Authors:** Irshad S. Sharafutdinov, Georgii D. Ozhegov, Alina E. Sabirova, Valentina V. Novikova, Svetlana A. Lisovskaya, Alsu M. Khabibrakhmanova, Almira R. Kurbangalieva, Mikhail I. Bogachev, Airat R. Kayumov

**Affiliations:** 1 Laboratory of Molecular Genetics of Microorganisms, Institute of Fundamental Medicine and Biology, Kazan Federal University, Kazan 420008, Russia; georgii_provisor@mail.ru (G.D.O.); alinka.zam@mail.ru (A.E.S.); 2 Industrial Drug Technology and Biotechnology, Perm State Pharmaceutical Academy, Perm 614990, Russia; 3 Department of Microbiology, Perm State Pharmaceutical Academy, Perm 614990, Russia; vvnperm@yandex.ru; 4 Kazan Scientific Research Institute of Epidemiology and Microbiology, Kazan 420015, Russia; s_lisovskaya@mail.ru; 5 Kazan State Medical University, Kazan 420012, Russia; 6 Biofunctional Chemistry Laboratory, Alexander Butlerov Institute of Chemistry, Kazan Federal University, Kazan 420008, Russia; alsu-khabibrakhmanova@mail.ru (A.M.K.); almira99@mail.ru (A.R.K.); 7 Radio Systems Department & Biomedical Engineering Research Centre, St. Petersburg Electrotechnical University, St. Petersburg 197376, Russia; rogex@yandex.com

**Keywords:** 2(5*H*)-furanones, biofilm, *Candida albicans*, drug resistance, synergy

## Abstract

The frequency of mycoses caused by drug-resistant fungal pathogen *Candida albicans* has increased drastically over the last two decades. The spread of drug-resistant strains, along with the limitations of currently available antifungals, complicates the management of fungal infections, thereby representing great challenges for clinical healthcare. Among various antimicrobial pharmacophores, 2(5*H*)-furanone derivatives have demonstrated antimicrobial, antifungal, and antibiofilm activities. In this study, we report the antifungal activity of the 2(5*H*)-furanone derivative **F105**, consisting of three pharmacophores, namely chlorinated 2(5*H*)-furanone, sulfonyl group, and *l*-menthol moiety. Although exhibiting moderate antifungal activity alone with the minimum inhibitory concentration (MIC) values of 32–256 μg/mL, **F105** potentiates the activity of fluconazole and terbinafine with fractional inhibitory concentration index (FICI) values of 0.27–0.50. Thus, 16 μg/mL of **F105** reduced the MICs of these antifungals against fluconazole-resistant *C. albicans* isolates four-fold, achieving similar values as for the intermediately susceptible phenotype. Confocal laser scanning microscopy revealed that the fluorescent 2(5*H*)-furanone derivative **F145** was also able to penetrate through biofilms formed by *C. albicans*. Indeed, in the presence of **F105**, even sub-MIC concentrations of both fluconazole and terbinafine led to significant reduction of *C. albicans* CFUs in the mature biofilm. Thus, **F105** appears to be a promising candidate for the development of novel antifungal agents as well as enhancers of current antifungal agents, particularly for the treatment of drug-resistant *C. albicans* infections.

## 1. Introduction

*Candida albicans,* commonly found in human microbiota, can under certain conditions cause a range of opportunistic diseases, especially in immunocompromised patients [1]. Thus, the overgrowth of *C. albicans* often leads to chronic infections of mouth [2], skin [3], or genitourinary tract [4] known as candidiasis.

Various treatment options for *C. albicans* infections are available to date, ranging from topical antifungal agents chlorohexidine and nystatin [5] to systemic drugs such as terbinafine, fluconazole, and novel echinocandins [6]. However, rising resistance of *Candida* to antifungals requires the development of new drugs [7,8]. Thus, even though no clinical breakpoints for terbinafine have been defined so far, terbinafine-resistant *C. albicans* clinical isolates have been widely reported [9,10]. Additionally, a triazole antifungal agent fluconazole, commonly prescribed for *Candida* infections, is unfortunately predisposed to causing the development acquired resistance since it exhibits only fungistatic activity [11].

Besides genetically determined resistance, *C. albicans* forms rigid biofilms where cells become phenotypically resistant to antimycotics and to the immune system of the host [12]. The extracellular polymeric substances of the biofilm matrix provide a diffusional barrier for the drug, thereby leading to low susceptibility of cells to antimycotics [13].

Amongst novel approaches targeting biofilm-associated infections, the 2(5*H*)-furanone derivatives have been suggested as inhibitors of the bacterial biofilm formation [14,15]. In particular, furanones have been shown to quench the bacterial quorum-sensing pathways in, for example, *Pseudomonas aeruginosa*, *Escherichia coli*, and *Staphylococcus* [14,16]. Some 2(5*H*)-furanone derivatives have also been reported to exhibit pronounced biocidal activity against biofilm-embedded *Staphylococcus aureus* [17], *S. epidermidis* [18], and *Bacillus cereus* [19]. Nevertheless, the effect of 2(5*H*)-furanone derivatives on fungal cells is less well understood to date. Halogenated 3-phenyl-5-acyloxymethyl derivatives of 2(5*H*)-furanone demonstrated antifungal activity against *C. albicans* with minimum inhibitory concentration (MIC) values ranging from 0.5 to 2 μg/mL [20]. However, fluconazole-resistant *C. albicans* demonstrated lower sensitivity characterized by MIC = 8 μg/mL. In another study, 3-arylidene-5-(4-chloro/ethyl-phenyl)-furanones exhibited both antibacterial and antifungal activity with MIC values of 12.5–50 μg/mL against both *C. albicans* and *S. aureus* ATCC-29737 [21]. The obvious disadvantage of furanones is their high toxicity against eukaryotic cells. In particular, the cytotoxicity concentration (CC_50_) values of active compounds are in the range of 1.5–32 μg/mL, thus exceeding the corresponding MICs only 2–8-fold [15,17,19,22,23,24] and thereby limiting their direct application to topical use only.

**Figure 1:**
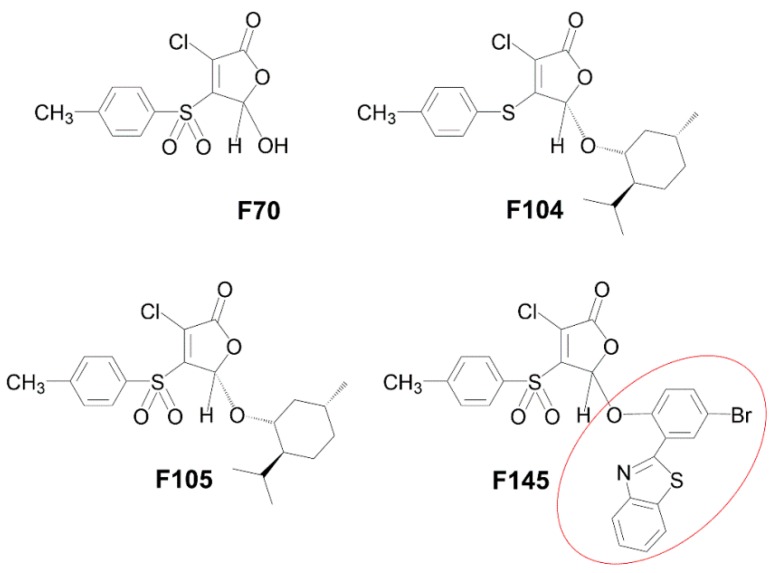
Molecular structures of 2(5*H*)-furanone derivatives **F70** [25], **F104 [17]**, **F105** [17], and **F145** [26]. The red circle in the structure of **F145** indicates a fluorescent 2-(benzothiazol-2-yl)-4-bromophenol (BTBP) moiety.

The aim of this work was to evaluate the antifungal activity of **F105** against a set of *C. albicans* isolates. We have investigated the antifungal activity of 3-chloro-5(*S*)-[(1*R*,2*S*,5*R*)-2-isopropyl-5-methylcyclohexyloxy]-4-[4-methylphenylsulfonyl]-2(5*H*)-furanone (**F105**) along with its analogues 3-chloro-5-hydroxy-4-[(4-methylphenylsulfonyl)]-2(5*H*)-furanone (**F70**) [25], 3-chloro-5(*S*)-[(1*R*,2*S*,5*R*)-2-isopropyl-5-methylcyclohexyloxy]-4-[4-methylphenylsulfanyl]-2(5*H*)-furanone (**F104**) [17], and the fluorescent 2(5*H*)-furanone derivative 5-[2-(benzothiazol-2-yl)-4-bromophenoxy]-3-chloro-4-[(4-methylphenyl)sulfonyl]-2(5*H*)-furanone (**F145**) [26] (see Figure 1 for structures). We found that **F105** was able to potentiate the antifungal activity of fluconazole and terbinafine against *C. albicans* cells, including resistant isolates.

## 2. Results

### 2.1. Antimycotic Activity of 2(5H)-Furanone Derivatives

#### 2.1.1. Susceptibility of *C. albicans* Isolates to **F105** and Conventional Antimycotics

The antimycotic activity of 2(5*H*)-furanone derivative **F105** carrying sulfonyl and *l*-menthol moieties was evaluated using a range of *C. albicans* clinical isolates (n = 26) and *C. albicans* NCTC^®^885-653 from the National Collection of Type Cultures (Perm, Russia) (Table 1). All clinical isolates were resistant to fluconazole (MIC ≥ 8 μg/mL according to M27-S4 guidelines prepared by the Clinical Laboratory Standards Institute (CLSI)), and moderately susceptible to terbinafine and nystatine [9,27]. **F105** completely inhibited the growth of various *C. albicans* isolates at concentrations of 32–256 μg/mL and higher, suggesting a weak antifungal activity. No antifungal activity was detected for other compounds **F70** and **F104**, lacking, respectively, sulfonyl or *l*-menthol moieties in comparison with **F105** (see Figure 1 for the structures). The compound **F145**, which in comparison to **F105** had a substitution of *l*-menthol moiety by fluorescent 2-(benzothiazol-2-yl)-4-bromophenol (BTBP), also demonstrated no antifungal activity. These data confirm that both sulfonyl and *l*-menthol moieties are strictly required for the antimycotic activity.

#### 2.1.2. **F105** Potentiated the Antifungal Activity of Fluconazole and Terbinafine against *C. albicans* Cells

**Figure 2:**
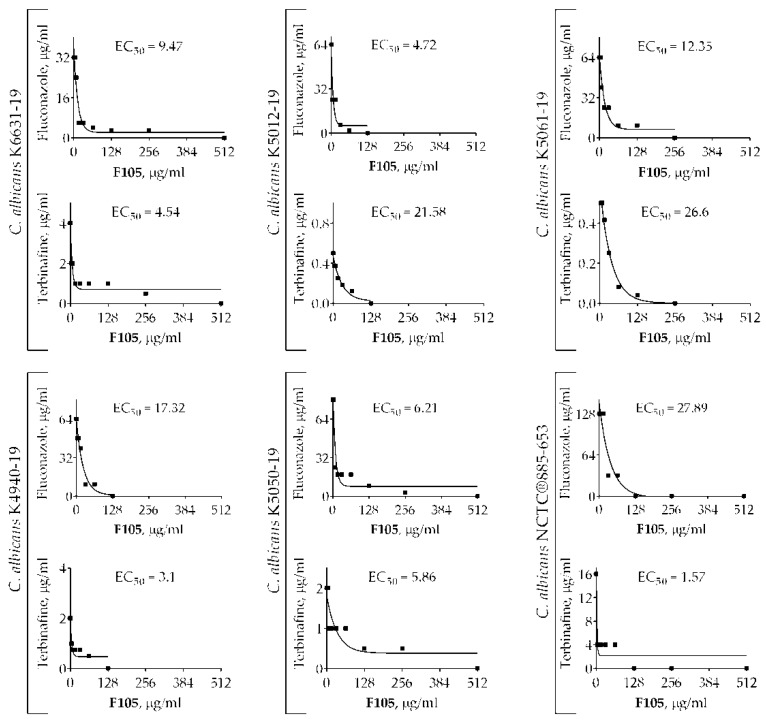
The EC_50_ values of **F105** leading to the 2-fold MIC reduction of fluconazole and terbinafine on different strains of *C. albicans*.

2(5*H*)-Furanone derivatives have been previously reported to increase the antimicrobial activity of antibiotics [17,19,24,28]. In particular, **F105** exhibits pronounced synergy with aminoglycosides (kanamycin, gentamycin, and amikacin) and benzalkonium chloride, significantly reducing the MICs of these compounds for *S. aureus* [17]. Therefore, we tested whether **F105** is capable of potentiating antifungal activities as well. *C. albicans* K6631-19, with a moderate susceptibility to all antimycotics, was chosen for this test (Table 1). While the molecular structure of nystatin contains a deoxysugar D-mycosamine, an aminoglycoside [29], the FICI_min_ value was 0.63 when combined **F105** with nystatin, suggesting no interaction (Table 2). In marked contrast, a pronounced synergy was observed for **F105** in combination with fluconazole and terbinafine, as their antifungal activity was considerably increased in the presence of **F105**. The checkerboard assay revealed FICI_min_ = 0.28 for both these antifungals in combination with **F105**. The calculated effective concentration (EC_50_) values of **F105** (determined as the concentration leading to the 2-fold reduction in MIC of either fluconazole or terbinafine) were 9.47 and 4.54 μg/mL, respectively (Figure 2, Table 2).

To confirm the synergistic effect of **F105** along with fluconazole and terbinafine, additional experiments were performed using *C. albicans* NCTC^®^885-653 and four *C. albicans* clinical isolates with various susceptibilities to antimycotics. The FICI values for **F105** and fluconazole revealed by the checkerboard assay ranged from 0.27 to 0.38, with corresponding EC_50_ values in the range of 4.72–27.89 μg/mL (Table 3). **F105** in combination with terbinafine exhibited FICI values in the range of 0.28–0.5 for five tested strains, suggesting a rather synergistic effect, while one strain showed FICI = 0.56. EC_50_ values remained within the range of 1.57–26.6 μg/mL. The obtained data suggest that **F105** in most cases potentiated the antimycotic activity of fluconazole and terbinafine against even resistant *C. albicans* cells (with the exception of *C. albicans* K5061-19 when **F105** was combined with terbinafine). Based on these data, a potential four-fold enhancement of the effect of both fluconazole and terbinafine in the presence of 16 μg/mL of **F105** could be expected. Thus, taking into account the rapid development of fluconazole-resistant strains among *C. albicans* isolates [30], **F105** could be a perspective candidate to reduce the MICs of conventionally used antifungals and maintain their efficacy for the treatment of drug-resistant fungi [8].

### 2.2. **F105** Enhanced the Capacity of Fluconazole and Terbinafine to Disturb C. albicans Plasma Membrane Integrity

The cell wall of *C. albicans* consists of an external protein coat and an internal skeletal layer [31,32], which protects the plasma membrane from damage and acts as a diffusional barrier for many antifungals [33,34]. The mechanism of antifungal activity of both fluconazole and terbinafine is based on disordering of the ergosterol synthesis pathway, an essential component of the *C. albicans* cytoplasmic membrane. Consequent changes in the plasma membrane permeability lead to fungal cell death. **F105** has been shown previously to directly interact with peptide molecules in this way, leading to the drop of the membrane potential of bacterial cells [17]. Therefore, we next analyzed the ability of **F105** to influence the membrane potential in *C. albicans* either alone or in combination with fluconazole and terbinafine.

**Figure 3:**
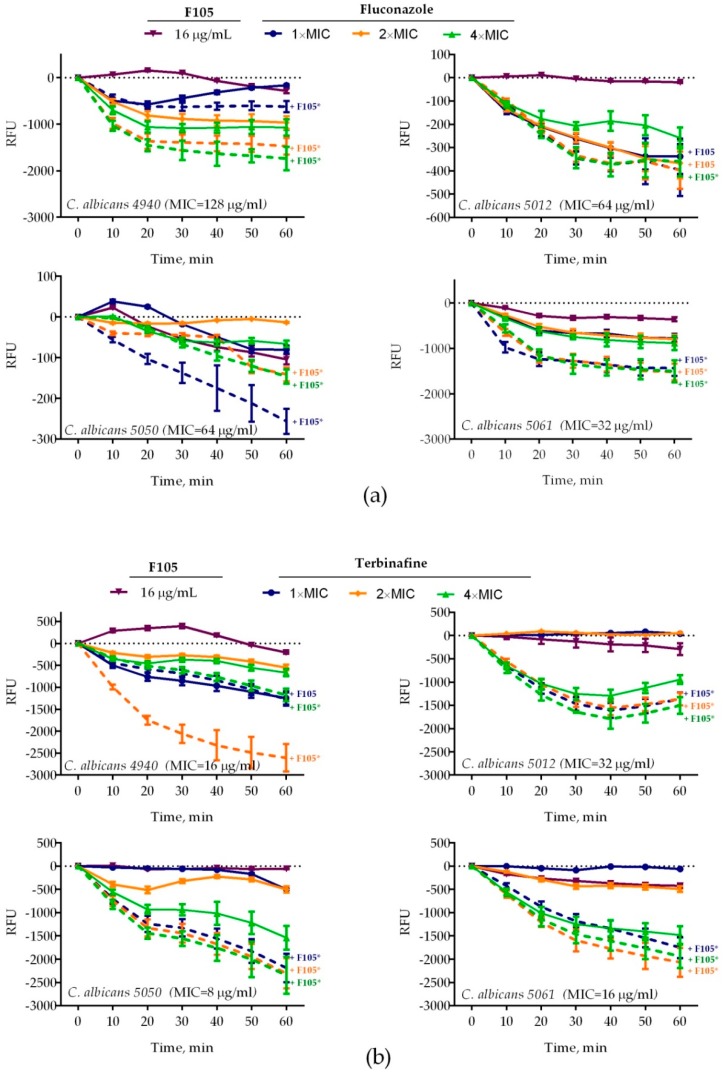
Relative membrane potentials of *C. albicans* isolates after treatment with **F105** 16 μg/mL and/or (**a**) fluconazole or (**b**) terbinafine (1×, 2×, or 4× of corresponding MIC, see Table 1 for values) either alone or in combination. *C. albicans* cells in the late exponential growth phase were harvested, washed with phosphate-buffered saline (PBS), and resuspended in PBS supplemented with 10 µM DioC_2_(3). After 30 min preincubation, compounds were added as indicated and the fluorescence was measured for 60 min with 10 min intervals. Lines represent the median values with IQRs from five independent measurements. In cases marked with *, the measured RFUs after 60 min of exposure were significantly lower in the presence of **F105** compared to similar treatments in the absence of **F105** according to the Kruskal–Wallis statistical test at *p* < 0.05. Corresponding MICs of antifungals are given in parentheses after the strain names.

**Figure 4:**
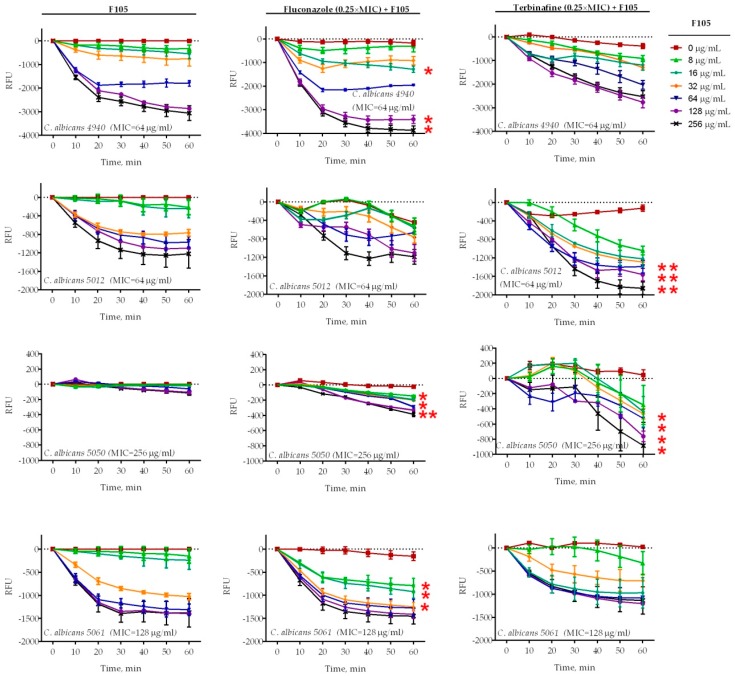
Relative membrane potentials of *C. albicans* isolates after treatment with various concentrations of **F105** and 0.25× MIC of fluconazole or terbinafine (see Table 1 for MIC values). *C. albicans* cells in the late exponential growth phase were harvested, washed with PBS, and resuspended in PBS supplemented with 10 µM DioC_2_(3). After 30 min preincubation, compounds were added as indicated and the fluorescence was measured for 60 min with 10 min intervals. Lines represent the median values with IQRs from five independent measurements. In cases marked with *, the measured relative fluorescence units (RFUs) after 60 min of exposure were significantly lower in the presence of **F105** compared to similar treatments in the absence of **F105** according to the Kruskal–Wallis statistical test at *p* < 0.05. Corresponding MICs for **F105** are given in parentheses after the strain names.

Four *C. albicans* clinical isolates (4940, 5012, 5050, 5061) exhibiting different susceptibilities to antifungals (see Table 1 for details) were treated with 16 μg/mL of **F105** and fluconazole (1×, 2×, or 4× of corresponding MIC) either alone or in combination. Treatment of *C. albicans* cells with 16 μg/mL of **F105** did not lead to a significant decrease in the membrane potential of any of the studied isolates (Figure 3). In marked contrast, the combination of **F105** with fluconazole led to a drastic decrease of the membrane potential in *C. albicans* 4940 and *C. albicans* 5061, while the effect was less pronounced in *C. albicans* 5012 and *C. albicans* 5050 (Figure 3a). These discrepancies in the susceptibility of different strains to **F105** and antifungals could be attributed to their particular resistance mechanisms by either efflux pump activation in *C. albicans* 4940 and *C. albicans* 5061, or ergosterol biosynthesis modification in *C. albicans* 5012 and *C. albicans* 5050. Remarkably, the combination of **F105** with terbinafine caused a sharp drop in the membrane potential in all four strains (Figure 3b). The observed effects confirmed the revealed synergy of fluconazole and terbinafine with **F105.** These data together with the observation of a 16-fold drop in **F105** FIC in the checkerboard assay allowed the speculation that *C. albicans* cells exhibit low permeability for **F105** itself. On the other hand, the membrane disorder caused by either fluconazole or terbinafine seemed to facilitate the penetration of **F105** into the cell. To test that assumption, the membrane potential was measured at sub-MIC concentrations of antifungals and various concentrations of **F105** (Figure 4). With the increase of **F105** concentration, the relative membrane potential decreased. In the presence of sub-MIC of fluconazole and furanone, a significantly steeper drop in membrane potential could be observed in *C. albicans* 4940, *C. albicans* 5012, and *C. albicans* 5061, although at high concentrations of **F105** only. In contrast, sub-MIC concentrations of terbinafine led to a faster loss of 3,3′-diethyloxacarbocyanine iodide (DioC_2_(3)) fluorescence only in *C. albicans* 5012 and *C. albicans* 5050 strains. The above also confirmed the furanone derivative’s cytotoxic effect, similarly to observations made in earlier studies [17,19,23,26]. The variations in observed speed of membrane potential drop could be attributed to the fact that measurements reflected the changes only 1 h after starting the treatment.

### 2.3. Penetration of Fluorescent 2(5H)-Furanone Derivative into C. albicans Cells

**Figure 5:**
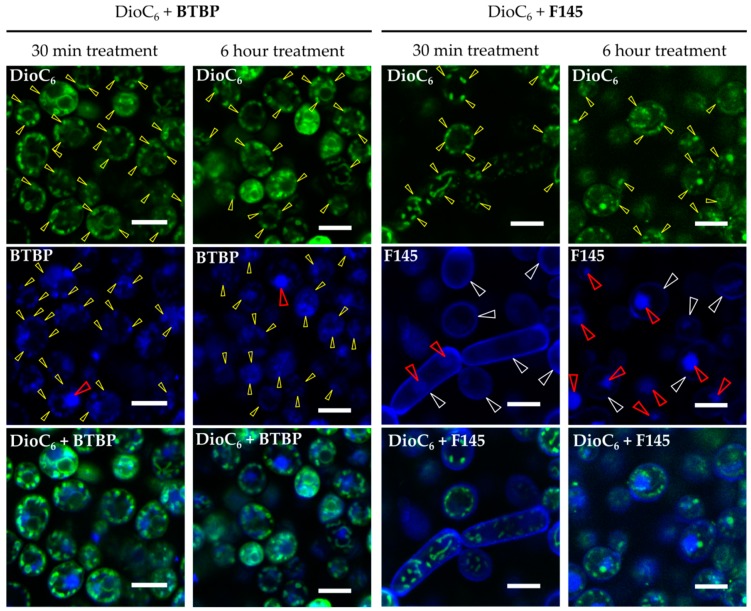
CLSM of *C. albicans* cells treated with DioC_6_(3) and **F145** for either 30 min or 6 h; control treated with DioC_2_(3) and **BTBP** fluorophore for 30 min or 6 h, respectively. White arrows indicate the stained cell wall, yellow arrows show the stained mitochondria, and red arrows indicate the stained nucleus. Scale bar is 5 μm.

The reduction of the **F105** FIC in the presence of either fluconazole or terbinafine allowed the suggestion that **F105** diffusion into the cell is complicated and is efficient only after membrane disturbance. To visualize the furanone penetration into *C. albicans* cells, the 2(5*H*)-furanone derivative **F145**, the fluorescent analogue of **F105**, was used. *C. albicans* cells were stained with both **F145** and 3,3′-dihexyloxacarbocyanine iodide (DioC_6_(3)) and analyzed using confocal laser scanning microscopy (CLSM). Since DioC_6_(3) is a membrane-potential-sensitive dye, the accumulation of DioC_6_(3) could be observed mainly in mitochondria and partially in the cytoplasmic membrane (Figure 5, yellow arrows). **F145** fluorescence was predominantly observed in the fungal cell wall after 30 min incubation (Figure 5). After 6 h incubation, strong **F145** fluorescence was mainly observed within the cell nucleus, suggesting its slow penetration through the cell wall. Interestingly, the blue fluorescence of **BTBP**, the fluorescent moiety of **F145**, was observed in mitochondria in overlay with DioC_6_(3) as well as in the nucleus after only 30 min of incubation. These data clearly indicate that **F145** binds initially with the cell wall of *C. albicans* and then slowly penetrates into the fungal cell and accumulates in its nucleus.

**Figure 6:**
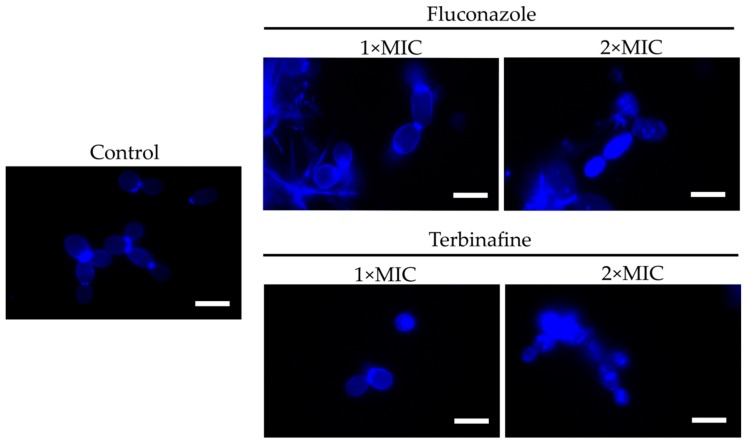
CLSM of *C. albicans* cells stained for 30 min with **F145** alone (control), or after 4 h pre-treatment with either fluconazole or terbinafine (1× or 2× MIC) as indicated. Scale bar is 2 μm.

To test the assumption that membrane disturbance facilitates the furanone derivative’s diffusion into the cell, *C. albicans* was treated for 4 h with either fluconazole or terbinafine (1× or 2× MIC) and then **F145** was added. After 30 min of incubation, the cells were analyzed with CLSM. In the presence of **F145** either alone or in combination with 1× MIC of the antimycotics, fluorescence was observed only in the cell wall (Figure 6). In contrast, in cells pre-treated with 2× MIC of either fluconazole or terbinafine followed by the **F145** addition, pronounced intracellular fluorescence could be observed, confirming that membrane disorder is required for the furanone derivative to penetrate into the cell.

**Figure 7:**
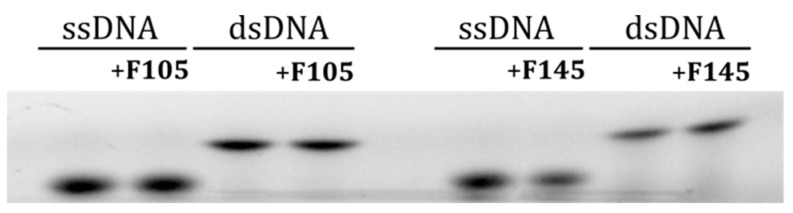
The electrophoretic mobility shift assay (EMSA) of 2(5*H*)-furanone derivative interactions with single- and double-stranded DNA.

Since the **F145** fluorescence was observed in the nucleus, we further analyzed whether furanone interacts with DNA in the cell nucleus. For that, both single- and double-stranded DNA fragments of 50 base pairs in size [35], each labeled with a Cy3 fluorophore, were mixed with either **F105** or **F145** and separated with 10% non-denaturing PAGE. No changes in the migration of either SS- or DS-DNA after furanone treatment could be observed, suggesting no interaction (Figure 7).

### 2.4. Penetration of Fluorescent 2(5H)-Furanone Derivative into C. albicans Biofilm

**Figure 8:**
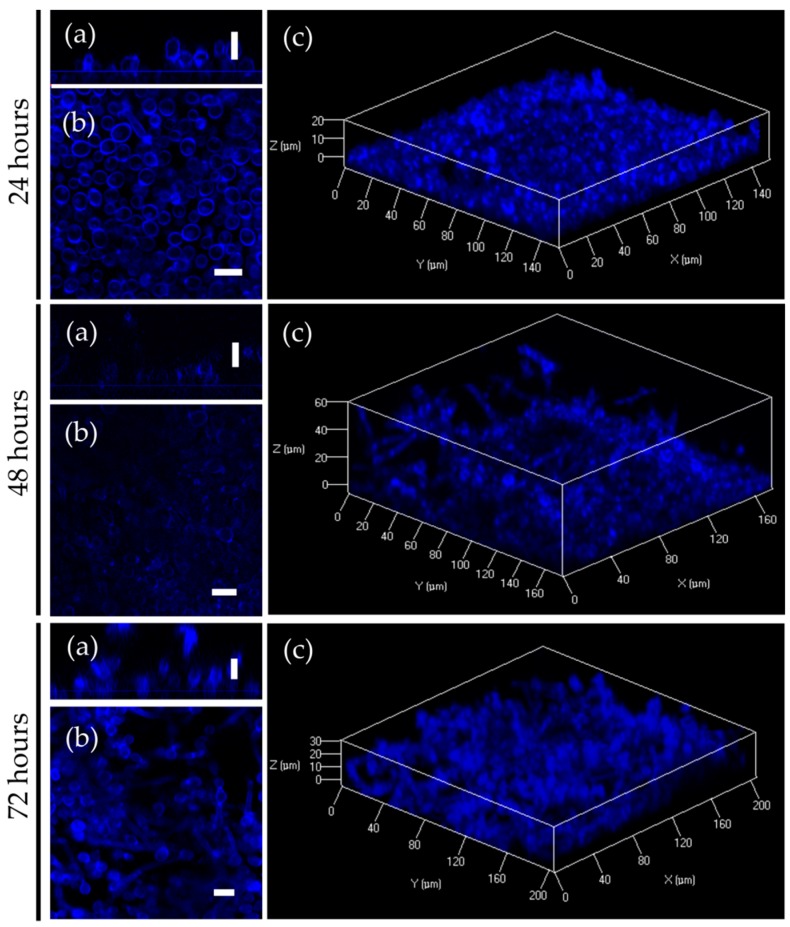
CLSM of *C. albicans* biofilm treated with **F145**. (**a**) X; Y orientation of the biofilm; (**b**) Z-stack of the biofilm; (**c**) 3D-model of the biofilm. Scale bar is 10 μm.

Biofilm formation represents one of the major virulence factors contributing to the pathogenesis of *C. albicans* infections. 2(5*H*)-furanone derivatives **F105** and **F145** have been previously shown to rapidly penetrate into the biofilm of *S. aureus* [17,26]. To investigate the diffusion ability of **F145** into the biofilm of *C. albicans*, the fungal cells were grown in RPMI broth for 24 h, 48 h, and 72 h, under static conditions to provide mature biofilm formation. **F145** was then added into the wells with established biofilm until the final concentration of 50 μg/mL was reached. After 1 h of incubation, the biofilm was analyzed with CLSM. The fluorescence of **F145** was observed throughout all layers of *C. albicans* biofilms of any age, indicating the penetration of the furanone through the biofilm matrix (Figure 8). Moreover, the fluorescent signal demonstrated a rather homogeneous distribution of **F145** through the biofilm with predominant accumulation in the cell walls of individual cells, including those located in the bottom layers. These data suggest that structural analogues of **F145** are capable of penetrating through the biofilm matrix of various pathogens.

**Figure 9:**
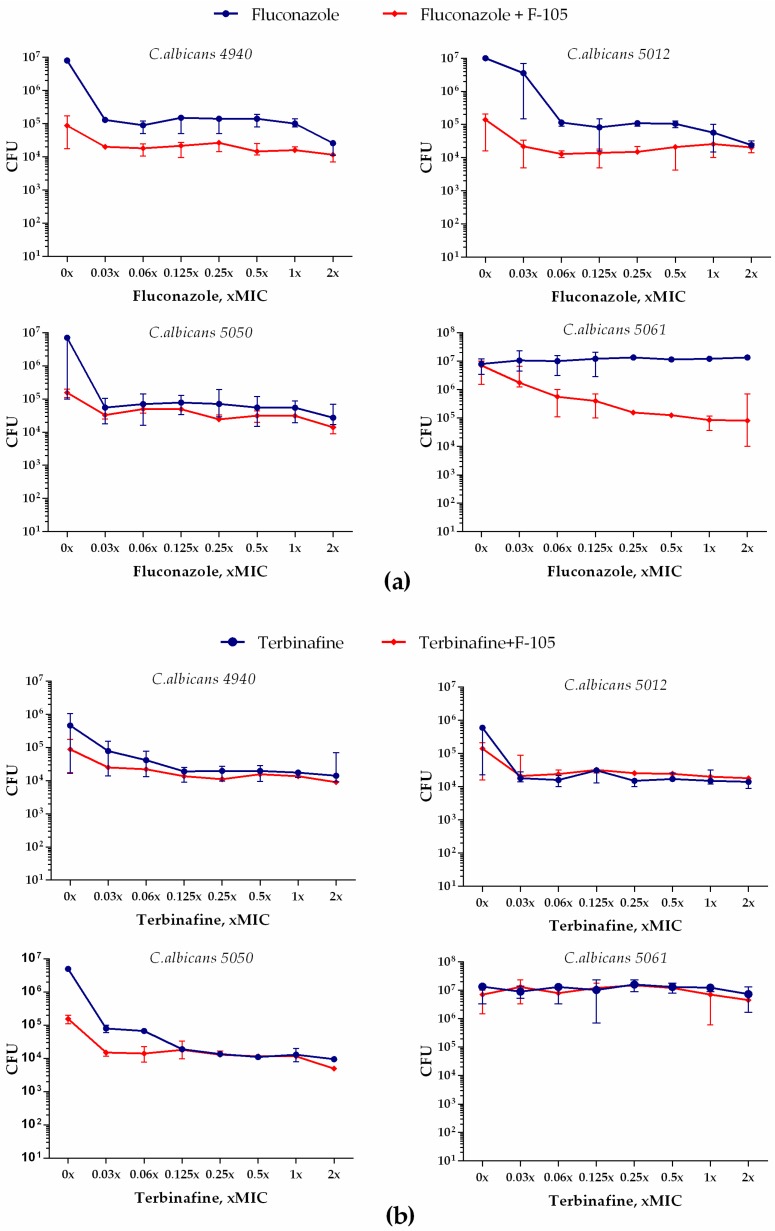
Antifungal effect of fluconazole (**a**) or terbinafine (**b**) alone and in combination with 16 μg/mL **F105** against *C. albicans* biofilm.

Next, we examined whether **F105** was able to potentiate the antifungal activity of fluconazole and terbinafine against mature 24 h old biofilms of *C. albicans*. Four *C. albicans* clinical isolates (4940, 5012, 5050, and 5061) were grown for 24 h to form biofilms. Different concentrations of either fluconazole or terbinafine were then added either alone or in combination with **F105** (in concentrations of 16 μg/mL) to the established *C. albicans* biofilm. After 24 h incubation, the CFU count was assessed by drop plate assay [17]. Even **F105** alone led to a two-log reduction in the CFU count in the biofilms of *C. albicans* strains 4940, 5012, and 5050 (see Figure 9, points 0×). The combination of **F105** with either fluconazole or terbinafine provided a further 10-fold decrease of the CFUs, while no complete death of the cells was observed (Figure 9). Taken together, these data suggest that even in combination with **F105**, fluconazole and terbinafine face the problem of the biofilm barrier, which provides antifungal resistance to *C. albicans*.

However, when **F105** and fluconazole were applied in combination, a 3-log reduction in the CFU counts was observed for the strains 4940 and 5012. Moreover, while the biofilm-embedded cells of the strain 5050 were not susceptible to fluconazole at the concentrations tested, in the presence of **F105**, a 2.5-log decrease of CFU count was achieved. The biofilm-embedded *C. albicans* 5061 cells were susceptible neither to **F105** nor to any of the antifungals even at the highest concentrations tested. Apparently, the biofilm matrix is not permeable for these antifungals, which is required for the facilitation of **F105** penetration into the cell. Nevertheless, fast penetration of **F145** into the biofilm allows its chemotype to be suggested for the development of novel systems to deliver antifungals into the biofilm, for example in the form of multi-pharmacophore drugs, to target biofilm-associated *C. albicans* infections.

## 3. Discussion

The rising frequency of candidiasis caused by *C. albicans* resistant to antimycotics highlights the spread of drug-resistant strains [10,30] and requires either the development of novel antifungals or the suggestion of alternative approaches to increase the activity of conventional ones. Therefore, the 2(5*H*)-furanone derivatives exhibiting both biocidal and antibiofilm activity against various bacteria could be of interest [17,19,24]. Here, we report an in vitro synergistic effect of the combination of terbinafine and fluconazole with the 2(5*H*)-furanone derivative **F105** for targeting *C. albicans* clinical isolates. The presence of **F105** lowered the MICs of both fluconazole and terbinafine approximately 4-fold (see Table 2 and Table 3). Although **F105** alone exhibited only moderate antimycotic activity in comparison with these antifungals, in their presence, its own MIC decreased, reaching relevant values of 2–4 μg/mL (see Table 1). The synergistic effects of the compounds were also confirmed by measuring the relative membrane potential (Figure 3 and Figure 4). Although the steepness of the DioC_2_(3) fluorescence drop varied significantly between strains, in general, in the combination of either fluconazole or terbinafine with **F105** this decrease was significantly faster, confirming rapid loss of energy supply to the cell. Thus, taking into account the rapid increase of fluconazole- and terbinafine-resistant *Candida* isolates, the reduction of active concentration of antimycotics could be useful for the treatment of *C. albicans* strains with intermediate susceptibility to antifungal drugs, and could contribute to the diversity to the currently limited antifungal arsenal.

The mechanism of synergy between **F105** and fluconazole or terbinafine seemed to be bilateral. Apparently, both fluconazole and terbinafine could affect the ergosterol composition in the fungal membrane due to the repression of squalene monooxygenase, which in turn can facilitate the penetration of **F105** into the cell. This assumption was supported by the fact that FIC of **F105** decreased up to 16-fold in the presence of either fluconazole or terbinafine, while the FICs of the latter exhibited a 4-fold decay only (see Table 2 and Table 3). Indeed, the permeability of the *C. albicans* membrane seemed to be low for the furanones. Thus, the fluorescent furanone derivative **F145** penetrated into the cell only after 6 h of treatment (Figure 5), although in the cells pre-treated with either fluconazole or terbinafine, intracellular fluorescence could be observed after only 30 min of incubation (Figure 6). These facts allow the speculation that **F105** targets intracellular proteins rather than the membrane, as has been shown previously for bacteria [26].

The molecular mechanism of the potentiation of either fluconazole or terbinafine by **F105** against *C. albicans* remains unclear. One could hypothesize that **F105** facilitates the penetration of small molecules of terbinafine (M = 291.43 g/mol) and fluconazole (M = 306.27 g/mol) into the cell by modification of the cell wall structure, while no corresponding effect could be observed for the larger molecules of nystatine (M = 926.09 g/mol). Another possible mechanism of antifungal activity of **F105** could be the disturbance of the biosynthetic apparatus of the cell through direct protein damage, since furanone derivatives can directly interact with a range of proteins both in vitro and in vivo [17,26].

Besides genetically determined resistance, *C. albicans* forms rigid biofilms which make the cell inaccessible to antifungals. Our results based on the fluorescent 2(5*H*)-furanone derivative (analogue of **F105**) allowed the conclusion that 2(5*H*)-furanone derivatives rapidly penetrate into *C. albicans* biofilm (Figure 8), although no complete death of biofilm-embedded yeast could be observed. This could be apparently attributed to the diffusional barrier effect of the biofilm matrix (Figure 9). Nevertheless, since the biofilm formation of *C. albicans* greatly contributes to its phenotypic drug resistance [13], the chemotypes of **F105** and **F145** seem to be interesting candidates for inclusion into complex multipharmacophore antifungals for biofilm-associated *C. albicans* infection treatment. Moreover, the 2(5*H*)-furanone derivative **F105** demonstrated a low risk of resistance development among *B. cereus* and *S. aureus* cells [19]. Considering the widespread occurrence of *C. albicans–S. aureus* mixed biofilms [36], the 2(5*H*)-derivative **F105** also represents an interesting candidate for targeting complex fungal–bacterial mixed infections.

## 4. Conclusions

In this work, we showed that 3-chloro-5(*S*)-[(1*R*,2*S*,5*R*)-2-isopropyl-5- methylcyclohexyloxy]-4-[4-methylphenylsulfonyl]-2(5*H*)-furanone (**F105**) exhibits pronounced antifungal activity in combination with either fluconazole or terbinafine against resistant *C. albicans* isolates. The relatively high toxicity of **F105**, with CC_50_ values in the range of 8–40 μg/mL for different cell lines [17], remains a limitation for its direct translation. Nevertheless, non-toxic concentrations of **F105** could increase the efficacy of fluconazole and terbinafine against resistant *C. albicans* strains. This allows the **F105** chemotype to be suggested as a promising starting point for the development of complex topical agents for targeting *C. albicans* infections, while further in vitro and in vivo studies are required.

## 5. Materials and Methods

### 5.1. Strains and Growth Conditions

*C. albicans* NCTC^®^885-653 from the National Collection of Type Cultures (Perm, Russia) and 26 clinical isolates (see Table 1 for the source) from the patients of Kazan Scientific Research Institute of Epidemiology and Microbiology (Kazan, Russia) obtained during 2019 were used for the experiments. Isolates were identified as *C. albicans* by using AuxaColor 2 Colorimetric sugar-assimilation yeast-identification kit (Bio-Rad) and confirmed via MALDI-TOF mass spectrometry (Bruker Biotyper system, Bruker Daltonics, Germany).

All strains were stored as a 50% glycerol stock at −80 °C and grown in RPMI broth. To obtain a mature biofilm, fungal cells were grown in 35 mm TC-treated culture plates under static conditions for 24, 48, or 72 h at 37 °C.

### 5.2. Tested Compounds

3-Chloro-5(*S*)-[(1*R*,2*S*,5*R*)-2-isopropyl-5-methylcyclohexyloxy]-4-[(4-methylphenyl)sulfonyl]-2(5*H*)-furanone (**F105**) [17], 3-chloro-5-hydroxy-4-[(4-methylphenylsulfonyl)]-2(5*H*)-furanone (**F70**) [25,37], 3-chloro-5(*S*)-[(1*R*,2*S*,5*R*)-2-isopropyl-5-methylcyclohexyloxy]-4-[4-methylphenylsulfanyl]- 2(5*H*)-furanone (**F104**) [17], the fluorescent 2(5*H*)-furanone derivative 5-[2-(benzothiazol-2-yl)-4-bromophenoxy]-3-chloro-4-[(4-methylphenyl)sulfonyl]-2(5*H*)-furanone (**F145**) [26] (see Figure 1 for the structures), and 2-(benzothiazol-2-yl)-4-bromophenol (**BTBP**) [38] were previously synthesized. The detailed synthesis as well as spectroscopic data can be found in References [17,25,26,37,38]. Three antifungal compounds, fluconazole, terbinafine, and nystatin, were used as reference antimycotics. All tested compounds were dissolved in DMSO until final concentrations of 10 mg/mL were reached for **F105**, **F145**, and fluconazole, or 5 mg/mL for terbinafine and nystatin.

### 5.3. Determination of Minimum Inhibitory Concentration (MIC)

The susceptibility of *C. albicans* to antifungal agents was assessed in accordance with the Clinical Laboratory Standards Institute (CLSI) standardized broth microdilution method. Measures of 100 µL of 2-fold serially diluted concentrations of antifungal agents in RPMI broth were mixed with 100 μL of *C. albicans* cell suspensions adjusted to 1–5× 10^5^ CFU/mL in 96 well plates and incubated at 35 °C for 24 h. The lowest concentration with at least 50% growth inhibition was determined as the minimum inhibitory concentration. Experiments were performed in biological triplicates with two technical repeats in each, and the medians were calculated.

### 5.4. Assessment of Synergy Between **F105** and Conventional Antifungal Agents

To assess a synergy between **F105** and antifungal agents, a checkerboard assay was performed as described previously [17]. Briefly, the final concentrations of both compounds ranged from 1/16 to 4×MIC for a furanone derivative and from 1/256 to 4×MIC for the antifungals. In total, nine dilution steps of antifungals and seven dilution steps of **F105** in RPMI broth were obtained. The microwell plates were incubated at 35 °C for 24 h. Each test was performed in triplicate and included a growth control without addition of any antifungal or **F105**. The fractional inhibitory concentration index (FICI) was counted from the concentrations in the first non-turbid well found in each row and column along the turbidity/non-turbidity interface, and the lowest FICI value was used to characterize the synergy. For the FICI interpretation, we referred to Reference [39]: FICI < 0.5 corresponds to synergy, 0.5 < FICI < 4 corresponds to either additive effects or indifference, while FICI > 4 corresponds to antagonism.

### 5.5. Membrane Potential Evaluation

Membrane potential was evaluated by detection of 3,3′-diethyloxacarbocyanine iodide (DioC_2_(3)) fluorescence. *C. albicans* cells were grown for 24 h, harvested, and washed with PBS. Cells were resuspended until a final density of 10^5^ CFU/mL was reached in PBS supplemented with DioC_2_(3) to a final concentration of 10 μM. After 30 min preincubation at 25 °C, **F105** and/or antifungals were added and the fluorescence was measured for 60 min with 10 min intervals using carboxyfluorescein (FAM) wavelength detection (the excitation and emission wavelengths were 497 and 520 nm, respectively).

### 5.6. Confocal Laser Scanning Microscopy

To assess the localization of **F145** within the biofilm and cells, the fungal cells and biofilms were treated with 2(5*H*)-furanone derivative **F145** for 60 min, with subsequent staining with DioC_6_(3) and propidium iodide. *C. albicans* cells and biofilms were analyzed under vital conditions using a direct confocal laser scanning microscope LSM700 (Carl Zeiss AG) at blue (405/410–508 nm), green (488/490–606 nm), and red (543/566–718 nm) channels. The obtained data were visualized using ZEN 12.0 software (Carl Zeiss Microscopy GmbH, Jena, Germany), with further quantification and statistical assessment using in-house developed software [40].

To evaluate whether furanone derivatives could penetrate the fungal cells, the 2(5*H*)-furanone derivative **F145** carrying a fluorescent 2-(benzothiazol-2-yl)-4-bromophenoxy moiety was used. 2-(Benzothiazol-2-yl)-4-bromophenol (**BTBP**) [38] itself was used as a control. Yeasts were grown for 24 h with agitation in Sabouraud broth, then washed and resuspended in PBS. **F145** or **BTBP** were added until the final concentration of 50 μg/mL and incubation followed for 30 min or 6 h. Cells were then stained with DioC_6_(3) and analyzed with CLSM.

### 5.7. Investigation of DNA-Binding Ability of **F105**

To assess the DNA-binding ability of F105, two 50 mer self-complementary primers corresponding to GlnR promoter sequence [35] were melted and annealed. A forward primer was labeled with cyanine3 (Cy3) on 5′ end. Measures of 10 nM of DNA were mixed with F105 (64 μg/mL final concentration) and pre-incubated at 37 °C in 1×TBE buffer in a final reaction volume of 10 μL. Samples were then separated on an 8% non-denaturing polyacrylamide gel and visualized on the Bio-RAD ChemiDoc XRS+.

## Figures and Tables

**Table 1:** Susceptibility of *Candida albicans* clinical isolates to **F105** and conventional antimycotics.

Isolate/Strain	Source	MIC (μg/mL)			
		F105	Fluconazole	Terbinafine	Nystatin
NCTC^®^885-653	NCTC Collection	64	64	8	8
K3836-19	Phlegm	64	32	4	4
K4026-19	Buccal swab	32	8	2	8
K4085-19	Buccal swab	256	16	4	4
K4146-19	Buccal swab	256	16	4	8
K46-19	Buccal swab	>256	128	8	8
K47-19	Buccal swab	256	64	32	16
K4940-19	Buccal swab	64	128	16	8
K4941-19	Buccal swab	>256	64	8	8
K4956-19	Buccal swab	>256	>128	16	4
K4978-19	Buccal swab	64	32	8	2
K5002-19	Cervical canal	>256	128	16	8
K5007-19	Buccal swab	64	>128	16	4
K5012-19	Buccal swab	64	64	32	8
K5014-19	Buccal swab	64	16	16	8
K5030-19	Buccal swab	32	128	16	8
K5038-19	Urethral mucosa	>256	32	8	16
K5050-19	Buccal swab	256	64	8	2
K5061-19	Buccal swab	128	32	16	4
K5077-19	Buccal swab	128	64	16	4
K5081-19	Buccal swab	128	64	32	4
K5092-19	Buccal swab	256	128	16	8
K5094-19	Buccal swab	>256	64	16	8
K5096-19	Buccal swab	32	64	16	2
K5097-19	Buccal swab	256	64	16	4
K625-19	Vaginal swab	256	32	4	4
K6631-19	Throat swab	>256	32	4	2

*The above data are medians calculated from a series of six independent measurements.

**Table 2:** Minimum inhibitory concentration (MIC), fractional inhibitory concentration (FIC), FICI, and (EC_50_) values of 2(5*H*)-furanone derivatives on *C. albicans* K6631-19.

	MIC	MIC (F105)	FIC	FIC (F105)	FICI_min_	EC_50_
Fluconazole	32	512	8	16	0.28	9.47
Nystatin	4	512	2	64	0.63	106.2
Terbinafine	4	512	1	16	0.28	4.54

**Table 3:** Synergistic effect of **F105** with fluconazole and terbinafine on *C. albicans* strains. MIC and FIC are expressed in μg/mL.

	***C. albicans* strain**	**MIC (F105)**	**MIC (FLC)**	**FIC (F105)**	**FIC (FLC)**	**FICI_min_**	**EC_50_ (F105)**
**F105 + Fluconazole (FLC)**	K6631-19	512	32	16	8	0.28	9.47
	K5012-19	64	64	16	4	0.31	4.72
	K5061-19	128	32	4	8	0.31	12.35
	K4940-19	64	128	16	16	0.38	17.32
	K5050-19	256	64	4	16	0.27	6.21
	NCTC^®^885-653	64	64	16	16	0.5	27.89
	***C. albicans* strain**	**MIC (F105)**	**MIC (TRB)**	**FIC (F105)**	**FIC (TRB)**	**FICI_min_**	**EC_50_ (F105)**
**F105 + Terbinafine (TRB)**	K6631-19	512	4	16	1	0.28	4.54
	K5012-19	64	32	16	8	0.5	21.58
	K5061-19	128	16	8	2	0.56	26.6
	K4940-19	64	16	4	4	0.31	3.1
	K5050-19	256	8	64	2	0.5	5.86
	NCTC^®^885-653	64	8	2	2	0.28	1.57
